# A Pilot Study of BRAIN BOOTCAMP, a Low-Intensity Intervention on Diet, Exercise, Cognitive Activity, and Social Interaction to Improve Older Adults’ Dementia Risk Scores

**DOI:** 10.14283/jpad.2024.104

**Published:** 2024-06-17

**Authors:** Joyce Siette, L. Dodds, K. Deckers, S. Köhler, I. Heger, P. Strutt, C. Johnco, V. Wuthrich, C. J. Armitage

**Affiliations:** 1 The MARCS Institute for Brain, Behaviour and Development, Western Sydney University, Sydney, New South Wales 2145 Australia; 2 Australian Institute of Health Innovation, Macquarie University, Sydney, New South Wales 2109 Australia; 3 Alzheimer Centrum Limburg, Department of Psychiatry and Neuropsychology, Mental Health and Neuroscience (MHeNs) Research Institute, Maastricht University, 6200 MD Maastricht, The Netherlands; 4 School of Psychological Sciences Macquarie University, Sydney, New South Wales 2109 Australia; 5 Macquarie University Lifespan Health and Wellbeing Research Centre, Macquarie University, Sydney, New South Wales 2109 Australia; 6 Manchester Centre for Health Psychology, University of Manchester, Manchester, M13 9PL UK; 7 Manchester Academic Health Science Centre, Manchester University NHS Foundation Trust, Manchester, M13 9PL UK; 8 NIHR Greater Manchester Patient Safety Research Collaboration, University of Manchester, Manchester, M13 9PL UK; 9 Level 6, 160 Hawkesbury Rd, Westmead, NSW 2109 Australia

**Keywords:** Brain health, dementia prevention, dementia literacy, lifestyle, multidomain intervention

## Abstract

**Background:**

Little is known about the impact of short, low-intensity multidomain dementia risk reduction interventions in older adults.

**Objectives:**

To examine the effectiveness and feasibility of a low-intensity multidomain lifestyle intervention on dementia risk and dementia literacy in Australian older adults.

**Design:**

Single-group pre-post design.

**Setting:**

Community-dwelling.

**Participants:**

A total of 853 older Australians (Mean age=73.3 years, SD=6.1) recruited from the community.

**Intervention:**

A 3-month dementia risk reduction program, BRAIN BOOTCAMP, including education, personalised risk information, physical cues for healthier choices and goal setting and planning to target four modifiable risk factors of diet, exercise, cognitive activity and social interaction in older adults.

**Measurements:**

The ‘LIfestyle for BRAin health’ (LIBRA) index was used to assess participants’ modifiable dementia risk based on 12 factors, with higher scores indicating greater risk. Dementia literacy was measured using a modified questionnaire derived from Dutch and British surveys, encompassing knowledge, risk reduction, and awareness aspects. Paired t-tests were used to compare dementia risk scores and dementia literacy before and after the program. Multivariate regressions were performed to identify sociodemographic and psychological factors associated with change in the LIBRA index.

**Results:**

Program attrition was high (58.3%). Participants who completed the program had decreased dementia risk scores (Cohen’s d=0.59, p<0.001), increased dementia literacy and awareness (Cohen’s d=0.64, p<0.001) and increased motivation to change lifestyle behaviors (Cohen’s d=0.25–0.52, p<0.016). Participants with higher motivational beliefs had greater dementia risk reduction.

**Conclusions:**

Improving older adults’ motivation and knowledge may help modify lifestyle behaviors to reduce dementia risk. However, program attrition remains a challenge, suggesting the need for strategies to enhance participant engagement and retention in such interventions.

## Introduction

**D**espite the evidence (1), there is still rudimentary understanding among the general public that dementia is a partly preventable disease, and many older adults are unaware of potential risk and protective factors (2). 45–70% of older adults are aware of the connection between modifiable factors and dementia risk, with significant knowledge gaps surrounding the contribution of metabolic and cardiovascular risk factors to dementia risk (e.g., diabetes and kidney disease), with greater knowledge gaps among specific subgroups (e.g., males, less formally educated) (3–5). To generate innovative initiatives that support meaningful behavior change across the population there is a need to adopt scalable implementation strategies and multifaceted lifestyle behavioral approaches (6, 7). These programs are built to be adaptable to different individual risk profiles and support sustainable behavior change (6). However, they can also be highly intensive, resource inefficient and methodologically challenging to carry out (8, 9). Evaluation of brief and lower-intensity education and intervention programs is needed to increase scalability of dementia prevention efforts.

Dementia prevention requires a multi-layered action plan (10) to tackle positive risk reduction through behavior change principles (11). Multiple large multidomain lifestyle trials have adopted various techniques (e.g., goal setting, shaping knowledge, feedback and monitoring) to successfully support risk reduction in middle to older aged adults with increased risk of dementia (e.g., Finnish Geriatric Intervention Study to Prevent Cognitive Impairment and Disability (FINGER) (12), French Multidomain Alzheimer Preventive Trial (MAPT) (13), Dutch Prevention of Dementia by Intensive Vascular Care (preDIVA) (14), European Healthy Aging Through Internet Counselling in the Elderly trial (HATICE) (15) and the AgeWell trial (16)). However, these intensive interventions are unlikely to be feasible, adaptable or translatable to diverse populations (17).

In an effort to advance dementia knowledge, the Dutch “We are the medicine ourselves” large-scale public health campaign demonstrated that adding pamphlets, physical items, educational booklets, and app-supported individualized feedback about one’s brain health prompted greater interest to increase literacy of dementia risk factors (4, 18). A similar 7-month public health campaign in Belgium resulted in more individuals (10.3%) believing dementia risk can be reduced, and improvements in the identification of 10 out of 12 modifiable factors (19), with exposure to the campaign associated with more awareness and willingness to take action to improve brain health (20). However, these studies have not yet investigated whether the observed improvements in awareness and knowledge translate into sustained behavior change or impacts on dementia risk in older adults. Additionally, understanding the mechanisms that underpin adherence and participation in behaviors towards dementia risk reduction could provide insight into targets for future interventions.

Therefore, a brief multidomain behavior change intervention called BRAIN BOOTCAMP was developed to increase dementia awareness and reduce dementia risk by encouraging behavior change in multiple risk factors (social interaction, physical activity, cognitive activity and diet) for older Australians (21). The program’s conceptual framework draws upon Michie et al.’s behavior change wheel (22), a comprehensive theoretical model that delineates core intervention functions and policy categories. These functions include education, emphasizing the dissemination of pertinent information; adding objects to the environment, focusing on modifications to the surrounding context to facilitate desired behaviors; and enablement, providing the necessary resources and support systems to empower individuals for behavior change (22).

By integrating these behavior change strategies, this program aims to prompt and sustain lifestyle modifications conducive to improved health and wellbeing. In this pilot study, we aimed to evaluate the impact of BRAIN BOOTCAMP on modifiable dementia risk and dementia literacy in community-dwelling older adults. Our specific objectives were to assess changes in modifiable dementia risk factors and improvements in dementia literacy within the participant group from pre- to post-intervention. The study hypothesized that engagement in BRAIN BOOTCAMP would lead to a significant reduction in modifiable dementia risk factors and an enhancement in dementia literacy compared to baseline measures.

## Methods

### Study Design

Full methods are described elsewhere (21). A single-group pre-post study design was used to evaluate the potential of a short (3-month), low-intensity multi-behavior change pilot intervention targeting dementia risk and dementia literacy in older adults.

### Participants

Participants were community-dwelling older adults residing in major cities and some inner regional areas of New South Wales, Australia. The inclusion criteria for this study encompassed community-dwelling individuals aged 65 years or older at the time of consent, literate in English, and possessing access to the Internet or the ability to request a hard copy for completing assessments. Furthermore, inclusion criteria for participants were intentionally designed to be inclusive of individuals with varying levels of risk factors, without imposing a minimum threshold, to explore the specific impact of individual risk factors on observed changes across demographic groups and ensure representation in our program. Exclusion criteria involved individuals with self-reported active major depression or an existing diagnosis of dementia, those unable to provide informed consent, or currently enrolled in any behavior change intervention.

Participants were recruited between January and March 2021 using a range of advertising methods, including flyers distributed at local medical clinics, print media and local radio advertisements. Interested candidates registered online and provided informed consent to participate in the program, complete online surveys at two time points (before and after the intervention) and to be contacted for an over-the-phone interview for further feedback about the program.

### Procedure

Once enrolled, participants were asked to complete their first online survey that included questions on dementia risk, dementia literacy, motivation to modify lifestyles that support dementia risk reduction, mental health, quality of life, and social networks. Additional questions covered basic demographics (e.g., age, gender, educational level) and medical history. Following completion of the first survey, participants received a BRAIN BOOTCAMP Box within 7 days containing resources and items for them to work through in the next three months (see Intervention below). The same survey was distributed at the end of three months. Participants did not receive any compensation for participation in the intervention.

### Outcomes

The primary outcome of the study was change in dementia risk scores from baseline to 3-months. Secondary outcomes included dementia literacy (belief that dementia is modifiable), dementia awareness (identification of risk and protective factors) and motivation.

#### Modifiable dementia risk score

Participants’ dementia risk was measured using the “LIfestyle for BRAin Health” (LIBRA) index, which is a weighted compound score of 12 modifiable risk and protective factors (e.g., smoking, physical inactivity, hypertension) that can be altered through risk management and lifestyle interventions (23). Each of these factors has an assigned weight based on the factor’s relative risk for dementia from published meta-analyses. Based on the presence or absence of a risk or protective factor, these weights are summed which results in the total LIBRA score. LIBRA scores range from −5.9 to +12.7 whereby higher scores indicate a greater risk of developing dementia (24). The LIBRA index has been extensively validated to predict cognitive decline, incident cognitive impairment, structural brain damage and dementia risk in various midlife and late life population-based studies (24–27). It has also been used as an outcome measure in multidomain intervention studies (28).

#### Dementia risk awareness and literacy

To measure dementia awareness and literacy, a modified questionnaire was employed. This questionnaire, derived from a Dutch public health campaign concentrating on enhancing awareness of dementia risk reduction (4), consists of 22 items. Among these, 10 items were adapted from the British Social Attitudes survey (29). Additionally, the questionnaire incudes sections covering self-reported dementia knowledge (e.g., How much would you say you know about dementia?), general statements on dementia risk reduction (e.g., Please state how much you agree or disagree with the statement. – There is nothing anyone can do to reduce their risk of getting dementia), personal experiences with dementia patients (e.g., Have you ever personally known anyone with dementia?), and awareness of the 12 modifiable dementia risk factors (30). This included items pertaining to physical activity, mental activity, diet, mental health, smoking, alcohol consumption, high blood pressure, diabetes, obesity, heart disease, kidney disease and cholesterol (4). Additionally, four sham factors (painkiller usage, personal hygiene, working in a noisy environment and having children) were included to assess monotone answering and statements. This original questionnaire has previously been administered in various populations to measure awareness and to evaluate the effect of public health campaigns focused on improving awareness of dementia risk reduction (18, 19, 31). In line with prior studies (e.g., 4, 5, 18, 31), when data were analysed, participants who reported their level of agreement as “strongly disagree” and “somewhat disagree” were grouped into a single “disagree” category; “neither disagree or agree” were coded as “neutral”, and reports on “strongly and somewhat agree” were grouped into an “agree” category.

#### Motivation

The 27-item Motivation to Change Lifestyle and Health Behaviors for Dementia Risk Reduction (MCLHB-DRR) tool was used to describe attitudes and beliefs associated with modifying lifestyle to facilitate dementia risk reduction (32). Items were distributed across seven subscales: perceived susceptibility (4 items), perceived severity (5 items), perceived benefits (4 items), perceived barriers (4 items), cues to action (4 items), general health motivation (4 items), and self-efficacy (2 items). The scale uses a 5-point Likert scale from 1 (strongly disagree) to 5 (strongly agree). Scores explore how factors may affect motivation to change lifestyle and health behaviors to reduce dementia risk (32). Internal reliability (*α* = 0.608–0.864) and test-retest reliability (*α* = 0.552–0.776) are moderate to high for all subscales for an Australian (32) and Turkish sample (33). Internal consistency was high in our sample (ranging from a = 0.910 (perceived barriers) to 0.985 (perceived susceptibility)).

### Intervention: BRAIN BOOTCAMP Program

The program comprised education, adding objects to the environment, and enablement, and was designed to increase people’s capabilities (e.g., knowledge), opportunities (e.g., restructuring the physical and social environment) and motivations (e.g., goal setting and planning) (22). In order to achieve this, after the completion of the baseline survey, participants obtained their BRAIN BOOTCAMP box which comprised:

1. personalized information based on their LIBRA dementia risk profile (e.g., detailing areas that are in favor of preserving brain health, areas that require lifestyle interventions to reduce their risk of dementia development, and areas for improvement). For instance, participants were provided with insights into their cognitive strengths and areas where improvement could be beneficial in terms of brain health. This aspect of the box aimed to educate participants about their individual risk factors, improve participants’ knowledge and awareness of dementia risk factors, and empower them to make informed decisions regarding their lifestyle choices to support brain health.
2. information booklet designed to improve dementia literacy and associated health behaviors (e.g., healthy eating and physical, social, and mental activities) (i.e., education). This booklet aimed to increase participants’ knowledge about brain health and dementia prevention strategies. These materials covered a range of topics, including the importance of physical activity, healthy eating habits, cognitive stimulation, social engagement, and stress management in maintaining optimal brain function and reducing dementia risk. By providing comprehensive education on these topics, the box aimed to equip participants with the knowledge and tools necessary to make proactive choices for brain health.
3. four physical cues that stimulate healthy brain habits (i.e., enablement). These items were designed to represent positive responses to modifiable factors such as physical inactivity (pedometer), cognitive inactivity (brain teaser flash cards), social isolation (social calendar), and poor diets (olive oil) to encourage individuals to adapt their lifestyle behaviors.
4. goal setting and planning also promoted individual action by enabling participants to strive for personal and social development using guided resources in the educational booklet and social calendar. During the intervention period, participants were also encouraged to set goals and self-monitor their behavior. This was made available in specific writing sections in the educational booklet as well as for each calendar month in the social calendar for each of the modifiable factors, e.g., “Your goal should be specific, measurable (use your pedometer), and be possible to achieve. Write your own goal here! I will try to take (number) steps a day”).

After six weeks, the research team contacted all participants by email to notify them of the halfway mark and reminded them to use the resources provided in the box.

### Statistical Analysis

All statistical analyses were conducted using SPSS version 27 and were carried out in accordance with an analysis plan developed prior to data extraction (21). First, we conducted a descriptive analysis of sociodemographic variables and outcomes to check for any significant differences between program completers and non-completers at the beginning of the study. Normal distribution of data was also assessed prior to analysis. Chi-squared tests or Fischer’s exact tests were performed for comparing dichotomous/categorical responses, and where normal distributions were not observed, Kruskal-Wallis tests were performed to test for differences between pre-and-post responses using nonparametric ranking methods. Cohen’s d effect size were defined as follows: a small effect size around 0.2, a medium effect size around 0.5, and a large effect size around 0.8.

To assess the efficacy of the program on dementia risk scores, paired t-tests were used to analyse changes between pre-and-post program results for each outcome. To adjust for potential confounding in the analysis of dementia risk change scores, we computed propensity scores (35) through logistic regression models and used them in subsequent analyses. Specifically, inverse probability weighting was employed to address potential biases arising from loss to follow-up. A predictive model was constructed to ascertain the likelihood of participants being lost to follow-up, with retention status (yes/no) at follow-up serving as the dependent variable. Baseline predictors, such as demographics, baseline LIBRA, motivational beliefs, were included as independent variables in this model. The resulting weights were inverted and incorporated into analytical models to adjust estimates back to the original sample at baseline. This approach aimed to mitigate selection bias resulting from the analysis of only approximately 40% of the initial sample, ensuring that individuals who were at high risk of dropping out but remained in the study at follow-up were appropriately accounted for in the analyses.

In a subsequent step, generalized linear models were used to investigate relationships of the primary outcome, changes in LIBRA scores (i.e., post minus pre scores), and demographic and motivational variables. The rationale for employing a change score in analyzing post-dementia risk scores lies in the empirical evidence suggesting that a residualized change model using post-intervention scores as the outcome is effectively equivalent to using the difference score as the outcome while adjusting for baseline dissimilarities (36). This approach also supports identification of underlying mechanisms and predictors of change in dementia risk scores over time. Thus, confounding variables and potential risk factors were individually assessed for their impact on LIBRA scores in the generalized linear model. These factors included age, gender, socioeconomic status based on the Index of Relative Socioeconomic Advantage and Disadvantage (IRSAD), categorized as low (1) and high (5) according to residential location, educational attainment (low, moderate, high), country of birth, and locality. Variables with unadjusted p-values exceeding 0.10, those exhibiting high rates of missing data, and those associated with elevated residuals were excluded from the final regression model (37). Omnibus tests were conducted to evaluate the overall significance of the models. The Benjamini-Hochberg procedure (38) was used to control the false discovery 15% rate, allowing for a more balanced adjustment in the presence of applied tests. The adjusted critical alpha level was p < 0.016.

## Results

### Baseline sample characteristics

**Figure 1:**
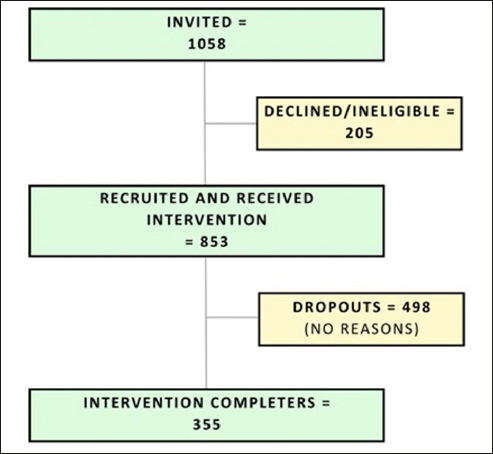
Flow chart of recruitment and completion of the intervention

A total of 1058 individuals accessed the registration link, of whom 853 were recruited after providing consent and completing the baseline survey. While all 1058 individuals initially expressed interest in participation, 205 did not proceed to complete the baseline survey. It is important to mention that eligibility for participation was contingent upon successful completion of the baseline survey (Figure 1).

Participants’ characteristics are provided in Table 1. 355 participants (41.6%) completed follow-up assessments and were considered program completers. There were statistically significant demographic differences between program completers and non-completers, where completers were more likely to be women, were born in an English-speaking country, and had higher levels of education (all p < 0.016) (Table 1). Completers also had significantly lower dementia risk scores and higher mean scores on all motivation subscales compared to non-completers (all p < 0.016) (Table 1). There were no differences between completers and non-completers on dementia literacy, quality of life, or social networks (all p > 0.016).

**Table 1:** Sociodemographic characteristics of participants who engaged in the program versus participants who dropped out

	**Drop-out (N=498) N (%)**	**Enrolled and completed (N=355) N (%)**	**χ**^**2**^** / F**	**p-value**
Gender				
Female	330 (66.3)	267 (75.2)	7.879	0.005*
Male	168 (33.7)	88 (24.8)		
Age (mean [SD])	73.4 [6.2]	73.2 [6.0]	0.213	0.644
65–69	154 (30.7)	122 (34.4)	5.262	0.154
70–79	269 (53.7)	175 (49.3)		
80+	88 (15.6)	55 (16.3)		
Country of birth				
English-speaking country	405 (81.0)	314 (88.5)	8.616	0.003*
Non-English speaking country	95 (19.0)	41 (11.5)		
Education				
Low	197 (40.1)	96 (27.0)	20.042	<0.001*
Intermediate	105 (21.4)	71 (20.0)		
High	189 (38.5)	188 (53.0)		
Socioeconomic status (quintile)				
1 (lowest)	26 (5.8)	17 (4.9)	7.607	0.107
2	74 (16.6)	51 (14.7)		
3	87 (19.6)	51 (14.7)		
4	57 (12.8)	38 (11.0)		
5 (highest)	201 (45.2)	190 (54.8)		
Locality				
Metropolitan	346 (76.0)	281 (79.8)	1.827	0.401
Regional	109 (23.9)	71 (21.2)		
Health condition				
Heart condition			2.089	0.554
Yes	125 (24.9)	90 (25.4)		
No	365 (72.7)	261 (73.5)		
Kidney disease			8.651	0.034
Yes	22 (4.4)	7 (2.0)		
No	472 (94.0)	346 (97.5)		
Diabetes			3.015	0.221
Yes	50 (10.0)	27 (7.6)		
No	447 (89.0)	327 (92.1)		
High cholesterol			4.542	0.209
Yes	264 (52.6)	181 (51.0)		
No	221 (44.0)	169 (47.6)		
High blood pressure			3.521	0.475
Yes	245 (48.8)	162 (45.6)		
No	246 (49.0)	187 (52.7)		
Smoking			1.7	0.429
Yes	11 (2.2)	355 (1.7)		
No	489 (97.4)	349 (98.3)		
Physical activity				
None	103 (20.5)	31 (8.7)	0.4	0.327
A little	191(38.0)	132 (37.2)		
Moderate	208 (41.4)	192 (54.1)		
Cognitive reserve (CRiQ) (mean [SD])	376.4 [167.2]	428.4 [150.9]	5.9	0.017
BMI (mean [SD])	26.9 (6.5)	26.6 (6.3)	0.3	0.556
Diet (MEDAS) (mean [SD])	10.3 (0.9)	10.4 (0.8)	0.1	0.748
Alcohol				
No alcohol intake	7 (1.4)	2 (0.6)	2.0	0.575
Up to 1 glass a day	386 (76.9)	280 (78.9)		
More than 1 glass a day	106 (21.1)	72 (20.3)		
Depression (PHQ-9) (mean [SD])	3.9 [4.0]	3.1 [3.2]	12.8	0.003*
Quality of life (EQ-5D-5L) (mean [SD])	0.8 [0.2]	0.4 [0.1]	5.6	0.018
Social networks (LSNS-6) (mean [SD])	16.1 [5.5]	16.9 [5.6]	0.1	0.888
Dementia risk (LIBRA index) (mean [SD])	−2.6 [2.0]	−3.0 [1.9]	7.3	0.007*
Dementia awareness (mean [SD])#	5.6 [3.2]	5.9 [3.1]	0.5	0.469
Dementia literacy##	322 (66.4)	250 (71.6)	2.6	0.108
Motivation (mean [SD])				
Perceived susceptibility	10.5 [4.2]	10.9 [3.4]	7.9	0.005
Perceived severity	14.3 [5.1]	15.5 [3.6]	14.3	0.001
Perceived benefits	13.9 [4.4]	14.9 [2.8]	16.3	<0.001*
Barriers	7.9 [3.4]	8.0 [2.6]	16.3	<0.001*
Cues	11.6 [4.4]	12.6 [2.9]	28.1	<0.001*
General health motivation	14.4 [4.8]	15.5 [2.6]	39.8	<0.001*
Self-efficacy	6.9 [2.4]	7.6 [1.4]	34.1	<0.001*

*Refers to significant at p < 0.016; #Refers to the number of identified risk factors; ##Dementia literacy refers to the proportion of respondents who agree that dementia is a modifiable condition.

The mean age of participants who completed the program was 73.2 years (SD=6.0) with the majority (75.2%) being female (Table 1). On average, participants had high levels of education (53%), were born in an English-speaking country (88.5%) and were living in metropolitan areas (79.8%). The following results refer solely to program completers.

### Primary outcome: Modifiable dementia risk score

Pre-and-post-program means of the modifiable dementia risk score (LIBRA), and motivation of enrolled participants are displayed in Table 2. Overall, participants showed a significant reduction in modifiable dementia risk levels post-program compared to pre-program (p < 0.001, Cohen’s d = 0.59, medium effect size). Participants who had higher general health motivation exhibited a significantly greater reduction in dementia risk score (β = 0.044, p < 0.001, 95% CI [0.028, 0.061]) (Table 3). Similarly, participants who perceived more benefits in dementia risk reduction had significantly higher change in dementia risk scores (β = −0.036, p < 0.001, 95% CI −0.047, −0.025]).

**Table 2:** Pre- and post-intervention means, confidence intervals, standard deviations, and effect sizes of enrolled participants (N=355)

**Metric**	**Pre**	**Post**	**t**	**p-value**^**a**^	**Cohen’s d**	**Interpretation**
	**Mean (SD)**	**Mean (SD)**				
Dementia risk score (LIBRA)	−3.00 (1.93)	−4.46 (1.50)	10.65	<0.001	0.59	medium
Motivation						
Perceived susceptibility	10.84 (3.27)	9.59 (3.39)	8.78	<0.001	0.43	medium
Perceived severity	15.49 (3.52)	14.96 (3.68)	3.35	<0.001	0.16	small
Perceived benefits	14.97 (2.66)	15.32 (3.14)	−2.21	0.028	-	-
Barriers	8.07 (2.60)	7.73 (2.87)	2.74	0.006	0.13	small
Cues	12.64 (2.89)	12.28 (3.40)	2.23	0.026	-	-
General health motivation	15.53 (2.39)	15.41 (2.90)	0.91	0.361	-	-
Self-efficacy	7.89 (1.38)	7.56 (1.59)	0.35	0.724	-	-
Dementia awareness	5.92 (3.10)	8.29 (3.26)	−5.194	<0.001	0.64	medium

a. P values in the mean column are calculated using 2-sided paired t-tests to compare means at the 2 time points.

**Table 3:** Summary of generalised linear regression analysis for predictors of change in dementia risk in 310 older adults

**Predictors**	**Change in LIBRA index# (N=310)**		
	**B##**	**95% CI**	p-value*
Intercept	1.320	−0.233–2.874	0.096
Gender			
Female	−0.125	−0.259–0.008	0.066
Male	reference		
Age group			
65–69	−0.062	−0.249-0.124	0.513
70–79	0.036	−0.131-0.203	0.669
80+	reference		
Country of birth			
English-speaking country	−0.172	−0.344–−0.00004	0.05
Non-English speaking country	reference		
Education			
Low	0.042	−0.088–0.173	0.527
Medium	0.035	−0.110–0.180	0.634
High	reference		
Socioeconomic status			
1 (lowest)	0.099	−0.150–0.347	0.438
2	0.065	−0.124–0.253	0.500
3	−0.029	−0.203–0.144	0.740
4	−0.003	−0.183–0.177	0.971
5 (highest)	reference		
Locality			
Metropolitan	−0.057	−0.232–0.118	0.525
Regional	reference		
Motivation			
Perceived susceptibility###	0.002	−0.007–0.010	0.740
Perceived severity###	−0.004	−0.018–0.010	0.611
Perceived benefits###	−0.036	−0.047–−0.025	<0.001*
Perceived barriers###	0.011	−0.005–0.026	0.180
Perceived cues to action###	0.007	−0.013–0.026	0.498
General health motivation###	0.044	0.028–0.061	<0.001*
Self-efficacy###	−0.025	−0.067–0.016	0.227

## computed as post-program LIBRA score minus pre-program LIBRA score, where a higher number indicates a greater reduction in dementia risk; ##refers to the unstandardized beta (B); ### computed by dichotomising responses (agree vs disagree). *Significant at p<0.016.

### Secondary outcome: Dementia literacy and awareness

**Figure 2:**
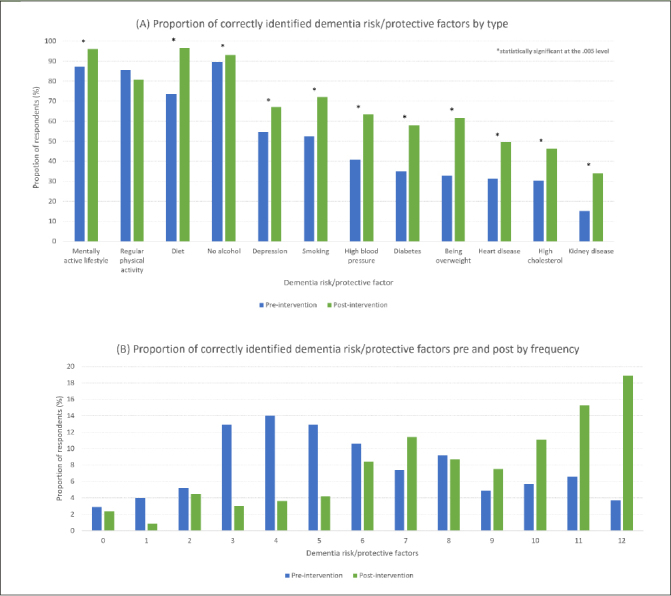
Proportion of correctly identified dementia risk/protective factors pre and post program by type (A) and frequency (B)

Most respondents (n=250, 71.6% of completer sample) reported that dementia risk could be reduced (i.e., dementia literacy) and close to half (48.1%) correctly identified six or more risk and protective factors preprogram entry (i.e., dementia awareness) (Figure 2). Following completion of the program, dementia risk literacy increased significantly, with an additional 10% of participants acknowledging it is possible to reduce the risk of dementia (71.6% at pre-program vs 81.4% at post-program, p < 0.001). Participants had significantly higher mean recognition of factors post program (pre: 5.9 vs post: 8.3, p < 0.001) indicating increased dementia awareness.

Figure 2 shows the percentage of identified risk and protective dementia factors pre- and post-program. At baseline, a cognitively active lifestyle was the most frequently identified factor (87.3%), followed by physical activity (85.6%), healthy diet (73.5%), and depression (54.6%). Vascular factors such as hypertension (37.3%), hypercholesterolemia (30.4%), and coronary heart disease (31.3%) were less recognized. Chronic kidney disease (15.2%) was the least identified factor. Following engagement in the program, dementia risk factor awareness increased for 11 factors, with the largest change for obesity (32.7% pre vs 61.7% post). There was no significant change in recognition of regular physical activity as a protective factor (p > 0.016), likely owing to its high identifiability at baseline already (87.3%).

### Secondary outcome: Motivation

Participants who completed the BRAIN BOOTCAMP program displayed significant changes in various health perception and motivation domains (Table 2). There was a substantial decrease in perceived susceptibility (Mean pre = 10.84, vs Mean post = 9.59; t = 8.78, p < 0.001, Cohen’s d = 0.43, medium effect size) and perceived severity (Mean pre = 15.49 vs Mean post = 14.96; t = 3.35, p < 0.001, Cohen’s d = 0.16, small effect size) of the condition following program completion. Additionally, participants had lower perceived potential barriers linked to altering lifestyle behavior to reduce risk post program (Mean pre = 8.07 vs Mean post = 7.73; t = 2.74; p = 0.006, Cohen’s d = 0.13, small effect size). There were no significant changes in participants’ reporting of perceptions regarding perceived benefits, cues to action (i.e., social influences to change lifestyle and health behavior for dementia reduction), general health motivation, self-efficacy and confidence in changing their lifestyle behavior post program (all p > 0.016).

## Discussion

Findings showed that participants who completed the program experienced reductions in dementia risk scores (medium effect), improved awareness of risk factors (small effect), reduced perceived susceptibility and severity of the condition (small to medium effect), and encountered fewer barriers to adopting brain healthy lifestyles (small effect). Individuals highly motivated to modify their risk and those perceiving more benefits from lifestyle changes showed the most significant reductions in dementia risk scores post-intervention, highlighting the potential impact of targeted motivation and education on dementia risk reduction.

While there is initial evidence suggesting the potential effectiveness of our intervention for individuals who actively engage with it, the high rates of dropout indicate a need for further investigation into factors influencing engagement levels. Dropout could be attributed to health, time, logistics, motivation-related reasons and demographic differences between completers and non-completers. It could also be that many participants are not willing to engage in this type of individual-level change, despite understanding how to lower their dementia risk which is thus a weakness of this type of intervention. This should be taken into consideration when interpreting the results as true effects of the intervention on all participants who enrolled at the beginning is expected to be considerably less significant. Policy-level change combined with a more tailored approach, for example with local stakeholder support (18), personalised and co-created intervention-boxes that varied across risk scores, gender and educational level, might have improved engagement (20).

Nevertheless, the triad of education, adding objects to the environment, and enablement may have a key role in reducing dementia risk by addressing motivational beliefs, and subsequent behaviour change among older adults. Education can help to empower individuals with knowledge about dementia risk factors, preventive measures, and lifestyle modifications, helping individuals to make informed health decisions and motivates the adoption of healthier behaviors, thus lowering the risk of developing dementia (39, 40). Enablement factors including reducing the perceived difficulty and increasing the simplicity of behaviors further complement these efforts by enabling individuals to initiate and sustain behavior changes. Furthermore, by providing resources, support systems, and brain training activities, this can enhance individual’s motivation for behavior change. This support enables individuals to overcome barriers and challenges, supporting improved cognitive function and reduced dementia risk (41).

Our results were positive in terms of dementia literacy and awareness. Dementia literacy (i.e., that dementia is modifiable) improved and knowledge of number of modifiable risk factors increased, although certain factors remain unchanged (e.g., physical exercise). Our sample’s dementia literacy was relatively high (71.6%) compared to the UK (47%) (42), the Netherlands (44%) (4), and Belgium (34.5%), and more or less similar to a Danish (66.5%) and Norwegian (70%) sample (19), all assessed using the same methodology. Noteworthy, the assessments in the UK, the Netherlands and Belgium all took place before 2018 so this difference could be due to a global rise in attention for the topic of dementia risk reduction. However we do note that we have a relatively small, highly self-selecting sample and these individuals may not be reflective of the samples who have been exposed to any dementia risk reduction campaigns. In those who completed our intervention, awareness increased even further after the intervention (10% increase), which may have supported changes in behavior. Indeed, prior studies recognize a positive relationship between the level of dementia awareness and lifestyle changes (4, 43, 44). Furthermore, Parial et al. identified that adults who engage in behavior change resulted in boosts of cognitive health (45). This prior work highlights that raising awareness on dementia risk reduction and consequential behavior change through more accessible methods of information to the public can be beneficial, particularly with a focus on more creative dissemination methods (46). More extensive and repeated sampling of the population will offer a more comprehensive insight into response rates and the extent to which population-wide risk reduction efforts have contributed to enhanced knowledge levels.

The observed decrease in perceived susceptibility and severity of dementia following the program aligns with previous literature highlighting the effectiveness of personalized interventions in altering risk perceptions for general health (49) and chronic conditions such as cancer (50, 51). These findings suggest that our program’s emphasis on providing individualized feedback based on participants’ dementia risk profiles can contribute to a more positive outlook regarding their risk of developing dementia. Previous research has shown that personalized feedback leads to greater understanding and acceptance of risk factors (49), empowers individuals to take control of their health (31, 52), increases awareness of potential consequences (53), and builds positive health behaviors (54). Future research should further examine the relative advantages of interventions incorporating personalized feedback compared to those lacking such feedback, aiming to identify the optimal approach.

The decrease in perceived potential barriers to behavior change post-program aligns with previous studies indicating the role of education and targeted strategies in overcoming barriers to health behavior modifications (55). Education increases knowledge about the benefits of behavior change (56) and equips individuals with problem-solving skills (57), while targeted strategies enhance self-efficacy (58) and leverage social support networks (59). However, the lack of significant change in self-efficacy post program contrasts with previous research showing improvements in self-efficacy following dementia literacy interventions (60). This discrepancy warrants further investigation into potential factors influencing participants’ confidence in their ability to make and sustain behavior changes.

Overall, these results support the notion that personalized, education-focused interventions can positively impact motivation domains related to dementia risk reduction. Future programs should consider incorporating such strategies to enhance self-efficacy alongside addressing cognitive and behavioral aspects to maximize effectiveness in promoting brain health and reducing dementia risk.

### Strengths and limitations

Our pre-post study design had no control group, which limits the findings to correlational relationships. It is also pertinent to contextualize our study’s findings within the broader landscape of health intervention acceptance and adoption. Whilst we applied attrition weights in the analyses to minimize bias due to selective dropout, the current sample mainly consisted of older adults who were highly motivated, highly educated or both, and rates of dropout were high. Therefore, selection bias may be present and the sample is an inaccurate representation of the general population, lowering external validity. Sampling techniques targeting people from less formal educational backgrounds and from other cultures will benefit the generalisability of these findings in future research. An additional limitation of our study is the use of change scores as the dependent variable. While this approach allows us to assess the effectiveness of interventions over time, it may also introduce variability due to individual differences in baseline scores and response patterns. Future research could explore alternative methods of analysis to mitigate these potential confounding factors and provide additional understanding of intervention outcomes.

### Implications

Our study showed that a short-term, population-targeted, multidomain intervention improved dementia literacy and dementia risk scores in program completers. Further work is required to better understand motivational factors influencing behavior change in older adults especially for participants who dropped out. This will reduce the ambiguity around cultures, values and behavioral risk factors and elucidate reasons for resistance to behavior change among some individuals. Specifically, investigating the specific barriers and challenges faced by older adults in sustaining engagement with brain health interventions can offer guidance for designing more targeted and effective approaches. Knowledge on risk and protective factors as well as other characteristics like health literacy, perceived relevance of the intervention, social support networks, and access to resources can be more thoroughly examined using mixed methods or open-ended questions to identify areas where interventions can be improved to better reflect population perspectives, especially regarding dropout reasons to enhance acceptability, and engagement.

Future studies should also aim to extend such interventions to explore potential long-term sustainable effects that do not perpetuate health inequalities in resource-constrained populations, a limitation of our study and other individual-level interventions (e.g., FINGER (12)). Our study also raises questions about the inclusivity of interventions and the potential for unintentional exclusion of individuals with lower educational backgrounds. Future research could further explore the design and delivery of interventions to better understand how educational differences may influence program effectiveness. However, an identified cultural and linguistic stigma associated with reducing dementia (47, 48) can be a barrier that can preclude people from seeking education about the disease. For this reason, it is imperative that awareness programs remain culturally and linguistically competent to allow for wider dissemination of dementia knowledge using an inclusive approach, to be able to address existing stigmas and lower brain health disparities.

## Conclusion

Our results suggest that a brief multidomain behavior change intervention has potential in reducing a modifiable dementia risk score and enhancing dementia awareness among older adults who successfully completed the intervention. However high dropout rates highlight the need to improve public engagement. Future research should focus on combining these individual-level targeted strategies with population-level interventions to create an environment that addresses barriers to participation and retention, and ultimately maximizes the intervention’s impact on dementia prevention and awareness.

## Data Availability

*Availability of data and materials:* Composite data can be made available upon reasonable request to the corresponding author (joyce.siette@westernsydney.edu.au).

## Notes

*Statement of Informed Consent:* All human subjects provided written informed consent prior to participation.
